# Chemical compositions, antimicrobial effects, and cytotoxicity of Asia minor wormwood (*Artemisia splendens* Willd*.*) growing in Iran

**DOI:** 10.1186/s13065-021-00759-w

**Published:** 2021-05-12

**Authors:** Fariba Heshmati Afshar, Masumeh Zadehkamand, Zahra Rezaei, Abbas Delazar, Vahideh Tarhriz, Parina Asgharian

**Affiliations:** 1 Biotechnology Research Center, Tabriz University of Medical Sciences, Tabriz, Iran; 2 Student Research Committee, Tabriz University of Medical Sciences, Tabriz, Iran; 3 Research institute of Bioscience and Biotechnology, University of Tabriz, Tabriz, Iran; 4 Drug Applied Research Center, Tabriz University of Medical Sciences, Tabriz, Iran; 5 Department of Pharmacognosy, Faculty of Pharmacy, Tabriz University of Medical Sciences, Tabriz, Iran; 6 Molecular Medicine Research Center, Biomedicine Institute, Tabriz University of Medical Sciences, Tabriz, Iran

**Keywords:** *Artemisia splendens*, Anti-proliferative activity, Antimicrobial activity, Cancerous cell lines, Flavonoids

## Abstract

**Background:**

Artemisia splendens from the Asteraceae family is a new source of biologically active compounds. The current study investigated to evaluate antimicrobial and cytotoxicity activity of methanolic extracts and their fractions obtained from aerial parts by agar disk diffusion and MTT methods, respectively. The active fractions were subjected to preparative HPLC for isolating the pure compounds, which were structurally elucidated, by ^1^H and ^13^C NMR.

**Results:**

The results showed that the methanolic extract and its 60% SPE fraction have the anti-proliferative activity on A549 cell line in comparison with the control group. Meanwhile, the methanolic extract and its 40% SPE fraction can inhibit the growth of Gram-positive strains as anti-microbial activity. The 60% SPE fraction also illustrated anti-proliferative activity on the HT-29 cell line compared to the control group. Chromatographic separations via preparative HPLC yielded 5 flavonoids and three flavonoid glycosides.

**Conclusion:**

Based on the results it can be concluded that *A. splendens* as a potential source of cytotoxic and antimicrobial compounds can be used in pharmaceutics.

## Introduction

The genus *Artemisia* is one of the largest and most important genera of the Asteraceae family used in traditional medicine worldwide [1]. Biologically active secondary metabolites such as phenolic compounds (flavonoids, phenylpropanoids, and coumarins), and terpenoids (sesquiterpene lactones) [2], which were isolated from plants of this genus, exhibited various pharmacological activities such as antimalarial, antitumor, antioxidant [3], anti-microbial [4], and anti-inflammatory [5]. It is also a large genus (with about 400 species), distributed in different parts of the world particularly in Europe, North America, Asia, and South Africa [1]. Thirty-four species of this genus are distributed in Iran. Among them, *Artemisia splendens* Wild. is commonly known as “Asia minor wormwood” and “Dermane derakhshan” in Persian. This species is one of the most popular herbs in traditional medicine and is mostly used for the treatment of diseases like malaria, hepatitis, cancers, and inflammations. Afshar et al. reported that the main volatile compounds of *A. splendens* were 1,8-cineol (4.7%), caryophyllene oxide (3.8%), valencene (3.5%) and α–terpinyl acetate (3.4%) [6]. In addition, Kazemi et al,. indicated that the major constituents of *A. splendens* were 1,8-cineole (14.5%), germacrene D (14.3%), α-pinene (11.3%) and bicyclogermacrene (11.3%)[7]. However, their chemical composition has not hitherto been described. Thus, the aim of this study was to analyze the chemical constituents of methanolic fractions obtained from the aerial parts of *A. splendens* to exert antimicrobial activity and cytotoxic effects on cancerous cells.

## Material and methods

### Plant material

*Artemisia splendens* aerial parts were collected from Arasbaran, at E: 31° 49′ 46″, N: 10° 49′ 38″ (altitude of 2470) East Azerbaijan Province, Iran in 2015. The plant material was identified by botanist Dr. Fatemeh Ebrahimi and the voucher specimen of the plant was deposited at the Herbarium of Faculty of Pharmacy, Tabriz University of Medical Sciences, Tabriz, Iran, under the registry code Tbz-FPh 717.

### Extraction

Air-dried aerial parts (100 g) were ground and extracted in a Soxhlet apparatus using MeOH. The extract was concentrated under the vacuum by a rotary evaporator at 45 °C and stored under refrigeration before analysis and biological evaluation.

### Fractionation of methanolic extract

Two grams of the MeOH extract were fractionated on Sep-Pak™ Vac 35 cc (10 g) C18 cartridge (Waters) eluting with a step gradient of MeOH-H_2_O mixtures (10:90, 20:80, 40:60, 60:40, 80:20, 100:0). A rotary evaporator was used to remove solvents at 45 °C under a vacuum. The procedure was repeated to increase the amount of SPE fractions.

### Isolation and identification of compounds

**Fig. 1:**
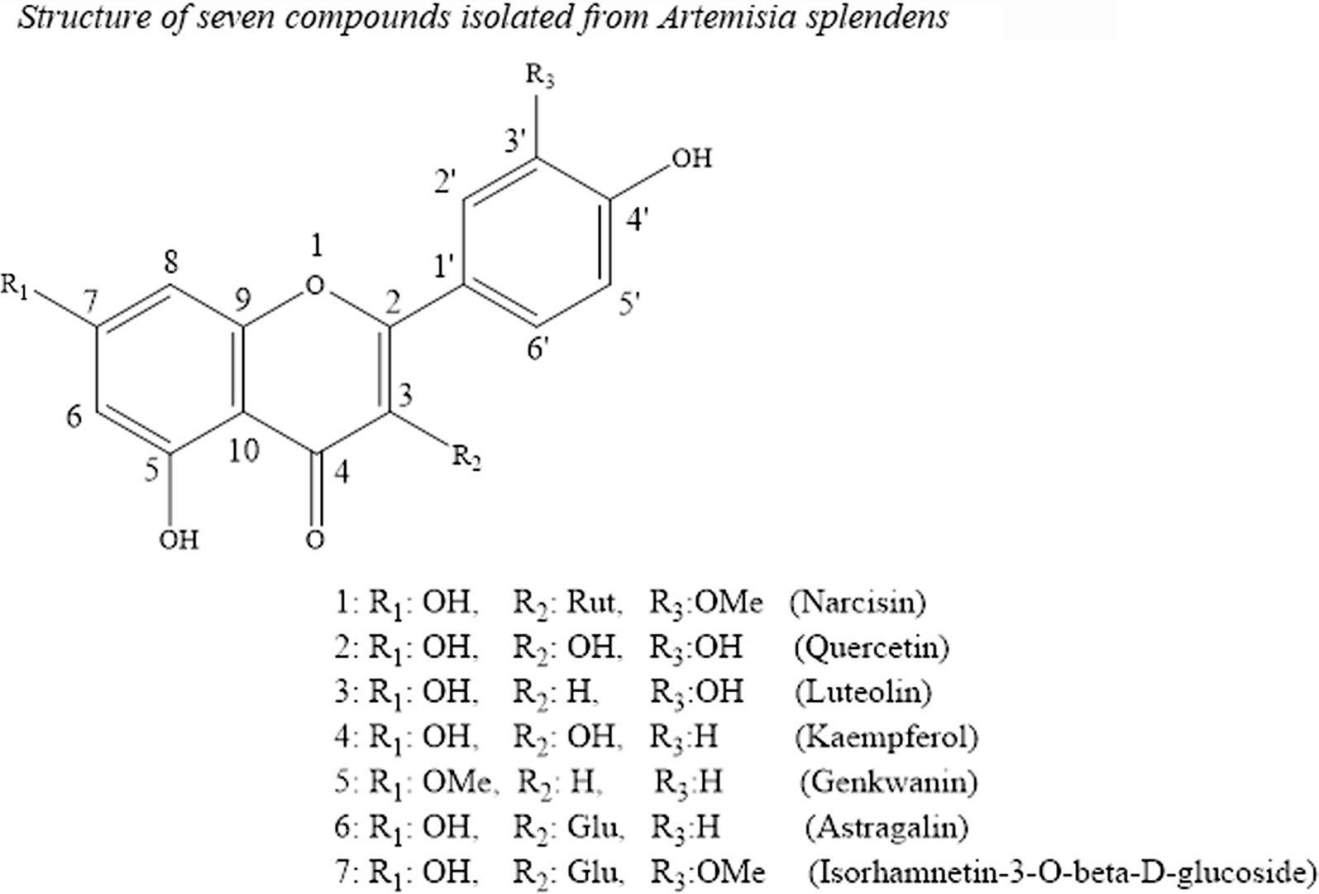
Structures of seven compounds isolated from *Artemisia splendens*

The pure compounds of (60:40) methanolic fractions were collected according to the method described by Afshar et al. [8], using preparative reversed-phase HPLC (Knauer, preparative pump 1800), with photodiode array detector (PDA) (Fig. 1). The flow rate was 8 ml/min and the injection volume was 1 ml. Furthermore, 40% SPE fraction was subjected to preparative reversed-phase HPLC using the following gradient elution: 0–30 min, linear gradient of 40–60% MeOH in water; 30–35 min, maintained in 60% MeOH in water; 35–38 min, linear gradient from 60 to 100% MeOH in water; 38–41 min, maintained in 100% MeOH; 41–43 min, linear gradient from 100 to 40% MeOH in water; 43–45 min, maintained in 40% MeOH in water; flow-rate: 8.0 mL/min and injection volume was 1 ml. Detection was done by a photo-diode-array detector (PDA) at 220 and 280 nm. This method allowed us to obtain astragalin [6], isorhamnetin-3-*O*-beta-d-glucoside [7] and narcisin [9]. Structures of compounds were elucidated by the literature data of respective compounds [3, 6–9]. Moreover, shift reagents in UV method were used [10] as follows: sodium methoxide (NaOMe) (Merck), aluminum chloride (AlCl_3_), aluminum chloride/ hydrochloric acid (AlCl_3_/HCl) (Merck) sodium acetate (NaOAc) and sodium acetate/boric acid (NaOAc/H_3_BO_3_) (Merck).

### Anti-proliferative activity

#### Cell culture

HT-29 (colon carcinoma cell line) and A549 (adenocarcinoma human alveolar basal epithelial cells) cell lines were prepared and cultured according to described method by Afshar et al. [11]. At 75% confluence, the cells were washed by phosphate-buffered saline (PBS) and cultured in 96-well plates (Nunc, Denmark) for MTT experiment.

#### MTT assay

The anti-cancer effect of the MeOH extract of *A. splendens* and its fractions were evaluated by MTT reduction colorimetric assay. The cells were cultured in a 96-well plate at a density of 1 × 10^4^ cells/well and incubated for 24 h. Cells were treated with different concentrations of extract and fractions which were prepared in dimethyl sulfoxide (DMSO). After 48 h of incubation, the medium was discarded and 3-(4,5-dimethylthiazol-2-yl)-2,5-diphenyltetrazolium bromide (MTT) solution (5 mg/ml in PBS) was added to each well and incubated at 37 °C and 5% CO_2_ for 4 h [12]. The medium was removed and then DMSO was added to dissolve purple insoluble formazan. The wells were shaken for 5 min and then the optical density of each well was determined at 570 nm wavelength by a microplate reader (ELISA plate reader, Bio Teck, Bad Friedrichshall, Germany). Each assay was accomplished three times for each cell line. Antiproliferative activity of extracts and their fractions were measured by relative viability according to the method described by Afshar et al. [11].

### Antimicrobial activity

#### Microbial strains

The following lyophilized forms of microbial cultures purchased from Pasture Institute, Tehran, Iran were used in this study: Gram-negative *Pseudomonas aeruginosa* (ATCC 9027) and *Escherichia coli* (ATCC 8739), Gram-positive *Staphylococcus epidermidis* (ATCC 12228), *Staphylococcus aureus* (ATCC 6538), and yeast *Candida albicans* (ATCC 10231).

#### Disc diffusion test

Activated microorganisms were transferred into Muller-Hinton broth medium (Merck, Germany) and incubated overnight at 37 °C. For providing an optical density of 0.5 McFarland (10^8^ CFU/mL as a standard optical density) of bacterial concentration, the centrifuged pellets (3000 rpm for 15 min) were washed twice and suspended in saline solution. The ultimate concentration of inocula was set up to 10^6^ CFU/mL with sterile saline solution. To achieve unified microbial growth, 10 mL of prepared inoculum suspensions were seeded in the autoclaved Muller-Hinton agar medium. Sterile discs (Whatman paper no. 6 mm diameter) located on the surface of media, were impregnated with 50 µL of different concentrations of extracts (1:1, 1:5, 1:10) which were dissolved in 50% aqueous DMSO. Then they were imbued with a 0.5 McFarland’s standard of mentioned bacteria covering 3 disks for test samples, one disk was used for negative control (including antimicrobial extracts and culture medium) and one disk was used for the positive control (including 0.5 McFarlend of the pathogen and culture medium). Afterward, for incubation and analysis, 100 µL of test solutions were poured into respective wells (200 mg/mL). The plates were transferred into the refrigerator to allow the diffusion of extracts approximately for 30 min, and then petri dishes were incubated at 37 °C for 24 h. Finally, the diameter of the inhibition zones obtained nearby each well (excluding well diameter), showing no bacterial growth, was measured with venire caliper. Doublet plates were prepared for each sample. Extracts and fractions displaying considerable anti-microbial effects were further evaluated for their minimum inhibitory concentration (MIC), which is the lowest concentration of each product for completely inhibition of the bacterial growth [13]. Successively, two-fold dilutions of MeOH extract and its fractions were utilized in broth. The pure sterile nutrient broth was used as control. MIC values were calculated after 24 h incubating the plates at 37 °C [14].

### Statistical analysis

All statistical analyses were done using Graph Pad Prism 8.01 and SigmaPlot 11 software. One Way ANOVA with a post hoc Tukey test was used for evaluating significant differences between groups. The experiments were performed in triplicates (n = 3) and all data were presented as mean ± S.D. *P* values < 0.05 which are regarded as significant.

## Results

In the present study, the pure compounds [1–7, 9] obtained from SPE fractions have been assayed. Moreover, anti-proliferative and anti-microbial activities of the MeOH extract and its different SPE fractions from aerial parts of *A. splendens* were determined and the results are indicated in Tables 1, 2. *(NMR spectroscopic data of compounds 1–8 (CD*_*3*_*OD, chemical shift δ in ppm, coupling constant J in Hz in parentheses):*

**Table 1:** Anti-proliferative activity of MeOH extract and its fractions against A549 and HT-29 cell lines

Samples	IC_50_ (μg/mL)	
	A549	HT-29
MeOH extract	617.72 ± 292.90	> 1000
10% SPE fraction	> 1000	> 1000
20% SPE fraction	> 1000	> 1000
40% SPE fraction	> 1000	> 1000
60% SPE fraction	98.63 ± 0.57	487.18 ± 38.06
80% SPE fraction	436.09 ± 194.17	> 1000
100% SPE fraction	> 1000	920.2 ± 62.50

**Table 2:** Anti-microbial activity of Methanolic extract and 40% MeOH–water SPE fraction of aerial parts of *A. splendens*

Samples		Microorganisms				
		*E. coli*	*P. aeruginosa*	*S. epidermidis*	*S. aureus*	*C. albicans*
Methanolic extract	DIZ (Mean ± SD) (mm)	–	–	12.5 ± 0. 7	28.5 ± 2.1	–
	MIC, mg/mL	–	–	200	100	–
40% MeOH–water SPE fraction	DIZ (Mean ± SD) (mm)	–	–	9.5 ± 0.70	11 ± 0.00	–
	MIC, mg/mL	–	–	–	–	–

**Compound 1:** ^1^HNMR and ^13^CNMR of the isorhamnetin-3-*O*-rutinoside was as follows: ^1^HNMR (200 MHz, CD3OD): Aglycone moiety: δ 7.95(1H, d, J = 2 Hz, H2′), δ 7.65 (1H, dd, J = 8.48,2 Hz, H6′), δ 6.91(1H, d, J = 8.48 Hz, H5′), δ 6.42 (1H, d, J = 2 Hz, H8), δ 6.22 (1H, d, J = 2 Hz, H6), δ 3.59 (3H, S, 3′-OMe). Glucose moiety: δ 5.24 (1H, d, J = 7.59 Hz, H1″), 3.2–3.6 (signal patterns unclear due to over lapping, H2″-3″-4″-5″), δ 3.90 (2H, H6″), δ 4.54 (l H, S, Hl‴), 3.2–3.6 (signal patterns unclear due to over lapping, H2‴-3‴-4‴-5‴) δ 1.16 (3H, d, J = 5.61 Hz, H 6‴). ^13^CNMR (50 MHz, CD_3_OD). Aglycone moiety: δ 177.5 (C4), δ 164.6 (C7), δ 162.1 (C5), δ 157.3 (C9), δ 156.8 (C2), δ 149.8 (C3′), δ 146.8 (C4′), δ 134.3 (C3), δ 122.7 (C6′), δ 121.3 (C1′), δ 113.4 (C5′), δ 115.3 (C2′), δ 104.8 (C10), δ 98.3 (C6), δ 93.5 (C8), Glucose moiety: δ 102.7 (C1″), δ 76.7 (C5″), δ 76.1 (C3″), δ 72.5 (C2″), δ 71.0 (C4″), δ 67.1 (C6″), δ 55.3 (OMe), δ 101.2 (C1‴), δ 74.5 (C4‴), δ 70.8 (C2‴), δ 70.5 (C3‴), δ 68.3 (C5‴), δ 16.5 (C6‴).

**Compound 2:** ^1^HNMR of quercetin was as follows: ^1^HNMR (200 MHz, CD3OD): δ 7.74(1H, d, J = 1.91 Hz, H2′), δ 7.66 (1H, dd, J = 8.40,1.91 Hz, H6′), δ 6.90 (1H, d, J = 8.40 Hz, H5′), δ 6.40 (1H, d, J = 1.5 Hz, H8), δ 6.18 (1H, d, J = 1.5 Hz, H6).

**Compound 3:** ^1^HNMR and ^13^CNMR of luteolin was as follows: ^1^HNMR (200 MHz, CD3OD): δ 7.38 (l H, d, H6′), δ 7.37 (1H, S, H 2′), δ 6.90 (1H, d, J = 8.92 Hz, H5′), δ 6.54(1H, S, H3), δ 6.44 (1H, S, H8), δ 6.21(1H, S, H6).^13^CNMR (50 MHz, CD_3_OD): δ 182.4 (C4), δ 164.7 (C7), δ 164.6 (C2), δ 162.1 (C5), δ 158.0 (C9), δ 104.1 (C10), δ 102.4 (C3), δ 98.7 (C6), δ 93.6 (C8), δ 149.6 (C4′), δ 145.6 (C3′), δ 122.2 (C6′), δ 118.9 (C1′), δ 115.3 (C5′), δ 112.7 (C2′). UV spectrum bands II and I respectively (MeOH, λ_max_, nm): 257, 368; + NaOMe 249, 405; + AlCl_3_ 272, 440; + AlCl_3_/HCl 268, 420; + NaOAc 270,390; + NaOAc/H_3_BO_3_ 262, 380. The UV spectrum of compound 5 with MeOH as a solvent is characteristic of flavones derivatives. Studying UV spectra data after the addition of NaOMe and production of 37 nm band I bathochromic shift is indicative of 4′-OH. After 5 min, no destruction of band I is showed that no moiety on C_3_ was observed. The addition of AlCl_3_ produced 72 nm band I bathochromic shift, so ortho di-hydroxyl structure of a compound can be elucidated and free 5-OH position of flavonoid is indicated as well. The addition of NaOAc and production of band II bathochromic shift of 13 nm indicated the free 7-OH position of flavonoid. After the addition of H_3_BO_3_, and production of 12 nm bathochromic shift on band I confirmed the presence of ortho di-hydroxyl structure.

**Compound 4:** ^1^HNMR and ^13^CNMR of kaempferol was as follows: HNMR (200 MHz, CD3OD): δ 8.10(2H, d, J = 8.70 Hz, H2′, 6′), δ 6.90 (2H, d, J = 8.70 Hz, H3′, 5′), δ 6.41(1H, d, J = 1.44 Hz, H8), δ 6.19 (1H, d, J = 1.83 Hz, H6). ^13^CNMR (50 MHz, CD3OD): δ 129.3 (C2′, 6′), δ 114.9 (C3′, 5′), δ 98.2 (C6), δ 93.1 (C8),

**Compound 5:** ^1^HNMR and ^13^CNMR of genkwanin was as follows: ^1^HNMR (200 MHz, CD3OD): 7.85(2H, d, J = 8.80 Hz, H2′, 6′), δ 6.93 (2H, d, J = 8.80 Hz, H3′, 5′), δ 6.66 (1H, S, H3), δ 6.48 (1H, d, J = 1.74 Hz, H8), δ 6.21 (1H, d, J = 1.74 Hz, H6), δ 3.97 (3H, S, OMe). ^13^CNMR (50 MHz, CD3OD): δ 164.0 (C7), δ 157.1 (C5), 128.0 (C2′, 6′), δ 115.6 (C3′, 5′), δ 103.8 (C3), δ 98.8 (C6), δ 94.4 (C8), δ 55.6 (C3 OMe). UV spectrum bands II and I respectively (MeOH, λ_max_, nm): 271, 350; + NaOMe 266, 405; + AlCl_3_ 276, 390; + AlCl_3_/HCl 278, 387; + NaOAc 273,363; + NaOAc/H_3_BO_3_ 270, 348. UV spectrum of compound 5 confirmed the structure of Genkwanin as well.

**Compound 6:** ^1^HNMR and ^13^CNMR of Astragaline was as follows: HNMR (200 MHz, CD3OD): δ 8.07 (2H, d, J = 8.81 Hz, H2′, 6′), δ 6.93 (2H, d, J = 8.81 Hz, H3′, 5′), δ 6.41(1H, d, J = 2.00 Hz, H8), δ 6.21 (1H, d, J = 2.00 Hz, H6). ^13^CNMR (50 MHz, CD3OD): δ 130.93 (C2′, 6′), δ 114.81 (C3′, 5′), δ 98.2 (C6), δ 93.1 (C8), δ 62.10- 76.40 (C6″–C2″).

**Compound 7:** ^1^HNMR and ^13^CNMR of Isorhamnetin-3-O-beta-D-glucoside was as follows: HNMR (200 MHz, CD3OD): δ 7.94 (1H, d, H2′), δ 7.59 (1H, d, J = 7.54 Hz, H6′), δ 6.91 (1H, d, J = 7.54 Hz, H5′), δ 6.41(1H, d, J = 2.00 Hz, H8), δ 6.21 (1H, d, J = 2.00 Hz, H6), δ 5.43 (1H, d, J = 6.35 Hz, H1″). δ 3–4 (5H, d, 5 H2″-H6″). ^13^CNMR (50 MHz, CD3OD): δ 178.80 (C4), δ 164.60 (C7), δ 158.80 (C2), δ 163.50 (C5), δ 160.00 (C9), δ 103.90 (C10), δ 130.90 (C3), δ 98.40 (C6), δ 93.30 (C8), δ 146.80 (C4′), δ 151.90 (C3′), δ 122.30 (C6′), δ 121.10 (C1′), δ 114.60 (C5′), δ 112.9 (C2′), δ 102.10 (C1″), δ 61.90- 72.90 (C6″–C2″).

**Compound 8:** This compound was named as Narcisin. The NMR data was the same as compound 1.

### Cytotoxic assay

In our study, the MeOH extract of *A. splendens* and its fractions were investigated for their cell growth inhibitory effect in human tumor cell lines such as HT-29 and A549 by MTT assay. The IC_50_ values of corresponding extract and fractions are reported in Table 1. The results indicated that the methanolic extract had weak to moderate anti-proliferative activity on the A549 cell line in comparison with DMSO control (*P* < 0.001) with an IC_50_ value of 617.72 ± 292.90 μg/mL. Whereas (10:90), (20:80), (40:60) MeOH: water SPE fractions did not affect the growth of A549 cells, 60% fraction inhibited cell growth of A549 cell line compared with control (*P* < 0.001) with an IC_50_ value of 98.63 μg/mL. In terms of colon cancer cell line, although, MeOH extract, could not decrease the amounts of cancer cells, its 60% SPE fraction significantly indicated anti-proliferative activity (*P* < 0.001).

### Anti-microbial activity

Among all the strains, only inhibition zones were observed against gram-positive *S. epidermidis* and *S. aureus* (Table 2). The MIC values for *S. epidermidis* was 200 mg/mL and for *S. aureus* 100 mg/mL which considered as very weak activity compared to the control by MeOH extract and 40% SPE fraction.

## Discussion

Cancer is one of the diseases with the highest mortality rate in developed countries and the second leading cause of death in developing countries [15]. Among all cancer types, lung and colon cancers are the most commonly diagnosed cancers in both genders [16]. Furthermore, due to the adverse effects of anti-microbial chemical drugs, the use of plants with anti-microbial effects is recommended to reduce side effects. Different studies have revealed that herbal medicines have anti-proliferative and anti-microbial activities through different functional mechanisms [17]. In the current study, the cytotoxic and anti-microbial effects of MeOH extract and its SPE fractions of *A. splendens* on A549 and HT-29 cells and different microorganisms have been investigated.

The results showed that 60% SPE fraction of MeOH extract had weak to moderate cytotoxic effect on both tumor cells in comparison with other fractions and MeOH extract as well as control (*P* < 0.001). It is worth mentioning that even though HT-29 was not influenced by MeOH extract, its 60% SPE fraction inhibited the growth of cells (*P* < 0.001). It can be concluded that the main anti-cancer ingredients of this extract have been accumulated in this fraction. Chromatographic separation of this fraction afforded 5 known flavonoids, namely isorhamnetin-3-O-rutinoside, quercetin, luteolin, kaemferol and genkwanin which are reported in this species for the first time. The anti-proliferative activity of various species of *Artemisia* has been evaluated by several researchers. These extracts inhibited the growth of different cell lines such as T47D, MCF-7 by inducing apoptosis [18]. Furthermore, previous studies have shown that quercetin and luteolin have cytotoxic effects on A549 with an IC_50_ value of 10.0 and 3.1 µM, respectively [19]. Luteolin induces apoptosis through the down-regulation of several apoptotic proteins in HT-29 [20]. It has been reported that kaempferol treatment promotes apoptosis of HT-29 human colon cancer cells through both extrinsic and intrinsic pathways [21]. Additionally, the IC_50_ value of genkwanin on A549 was determined as 9 µM [22]. A combination of solid-phase-extraction (SPE) and reversed-phase prep-HPLC analysis of the aerial parts of the SPE fraction (60%) afforded five flavonoids, which were isorhamnetin-3-O-rutinoside, quercetin, luteolin, kaempferol, and genkwanin. Structural identification of isolated phytochemicals was carried out by UV and NMR analysis and comparison of the data with the reported values. According to our knowledge, none of these phenolic compounds were isolated from *A. splendens.* Although in prior studies some compounds such as melilotoside have been reported from this plant [23].

The 1H-NMR spectrum of compound 1, revealed a doublet resonance at δ 7.95 (1H, d, 2.0) was specified for H-2′. The doublets at 7.65 (J = 1.70, 8.40 Hz) and 6.91 (J = 8.40 Hz) indicated ortho-coupled aromatic H-atoms assignable to H (6′) and H (5′), respectively. Besides, doublet resonances at δ 6.22 (1H, 2.0) and δ 6.42 (1H, 2.0) for the H (6) and H (8), indicated the meta coupled connection. Furthermore, doublet resonance at δ 5.24 showed the presence of an anomeric proton of glucose. The presence of singlet resonance at δ 4.54 and doublet resonance at δ 1.11(J = 6.04 Hz) is indicative of anomeric proton and CH_3_ (C_6_) of rhamnose, respectively. Mentioned data are consistent with the isorhamnetin-3-O-rutinoside (Narcissin) structure which has been proved by the previously published papers. The presence of this compound has been observed in other species of *Artemisia* such as *A. sublessingiana, A. absinthium, A. vulgaris,* and *A. incana* [24, 25].

In terms of compound 2 (quercetin), the UV spectrum of this compound indicated the presence of OH-4′ and 3 moieties due to bathochromic shift of band I and destruction of it after 5 min. The addition of AlCl_3_ reagent and observing a bathochromic shift in band I has been proved the presence of sensitive and resistant moieties of this structure. Subsequently, by adding HCL, the hypsochromic shift compared to the AlCl_3_ graph as well as a bathochromic shift to the MeOH graph illustrated the existence of both sensitive moieties (ortho dihydroxy of 3′, 4′) and resistant ones (free 3-OH and 5-OH). Furthermore, ^1^H and ^13^C-NMR spectrum have been proved this structure as well. All of the mentioned data are consistent with many previous investigations as well [26]. Spectrum data of compound 4, completely are in line with compound 2, except little difference in B ring with a symmetric structure in which the spectrum data are in line with published papers [27]. In the case of compounds (3) and (5), UV spectra were identical with flavone structure, (which was supported by ^1^H-NMR and ^13^C-NMR spectrums, showing characteristic signals appeared at δ 6.54 (1H, s, H-3), 6.66 (1H, s, H-3), luteolin, and genkwanin, respectively [26, 28].

MeOH extract and 40% SPE fraction of this plant showed inhibition zones against *S. epidermidis* and *S. aureus*. Although the MIC values for these pathogens are 100 and 200 mg/ml which considered as weak to inactive. It seems that it can be achieved potent anti-microbial agents via more isolating compounds from MeOH extract and 40% SPE fraction. Furthermore, in extract and fractions due to there are broad ranges of compounds, different biological activity might be observed (because the compounds have synergist or antagonist activity with each other). Previous studies showed MeOH extract of *A. nilagirica* indicated inhibitory activity against Gram-positive, as well as Gram-negative with DIZ value of 12 mm [29]. Furthermore, the MeOH extract of *A. nilagirica* was active against *M. smegmatis* [30]. Buffered methanol (80% methanol and 20% PBS) and acetone extracted substances from *A. absinthium* were active against *E. coli*, *S. infantis*, *S. aureus,* and *L. monocytogenes* [31]. *A. capillaris* Thunb. and *A. caruifolia* Buch. demonstrated inhibitory activity against *B. cereus* and *L. monocytogenes* [32]. *A. annua* and *A. vulgaris* L. illustrated the most prominent anti-bacterial activity against *S. mutans* [33].

To the best of our knowledge, no data were available on the antimicrobial activity of *A. splendens* MeOH extract. 40% SPE fraction for bearing moderate inhibitory effect on microorganisms was selected for further investigation using prep-HPLC afforded three main compounds as following structure. Compound 6 was isolated as yellow needles. HRESIMS analyses (positive and negative ion modes) of compound 1 revealed the *pseudo*-molecular ions at m/z 471 [M+Na]^+^ and 447 [M−H]^−^, which was in agreement with the molecular formula C_21_H_20_O_11_Na and C_21_H_19_O_11_, respectively. The ^1^H-NMR spectrum of compound 1 revealed the doublets resonance at δ 8.07 (2H, d, J = 8.81 Hz) and δ 6.93 (2H, d, J = 8.81 Hz) were specified for H-2′, H-6′ and H-3′, H-5′, respectively. In addition, doublet resonances at δ 6.41 (1H, d, J = 2.00 Hz) and δ 6.21 (1H, d, J = 2.00 Hz) for the H-8 and H-6, indicated the meta coupled connections, correspondingly. The above-mentioned data suggesting the presence of a flavonoid structure with a para pattern in the B ring. In addition, the ^1^H-NMR spectra of compound 6 confirmed the presence of an overlapping signal for five protons (δ 3–4 ppm) and an anomeric proton (δ 5.45, 1H, d, J = 7.48 Hz) corresponding to a glucosyl moiety with β configuration. The UV spectrum of compound 1 with MeOH as a solvent is characteristic of flavonols derivatives. Studying UV spectra data after the addition of NaOMe and production of 60 nm band I bathochromic shift is indicative of 4′-OH. Remaining of band I after 5 min indicated that C-3 has substitution except for OH. The addition of AlCl_3_/HCl did not cause any hypsochromic shift in the AlCl_3_ spectrum. Therefore, it can be concluded there is no ortho-dihydroxy group (3′ and 4′-OH) in the B ring. Furthermore, it leads to the presence of OH in C-5. Addition of NaOAc and production of band II bathochromic shift of 7 nm indicative of free 7-OH. In studying the ^13^C-NMR, the presence of four signals at δ 60–75, confirms the presence of glucose in the composition. Moreover, signals at δ 130.93 and 114.81 corresponded to C-2′, C-6′ and C-3′, C-5′, respectively. Thus, compound 6 was identified as Astragalin. All spectroscopic data were in good agreement with the literature data of respective compounds [34, 35]. While Astragalin [6] was previously reported from *A. transiliensis* [36] and *A. annua*, [3] this is the first report on the presence of Astragalin in the *A. splendens*.

Compound 7 was isolated as a yellow amorphous powder. ESIMS and FABMS analyses (negative and positive ion modes) of compound 7 revealed the *pseudo*-molecular ions at m/z 477 [M−H]^−^ and 479 [M+H]^+^, which was in agreement with the molecular formula C_22_H_21_O_12_ and C_22_H_23_O_12_, respectively. The ^1^H-NMR spectrum of compound 7, revealed a singlet resonance at δ 7.94 (1H, s) was specified for H-2′. The doublets at δ 7.59 (1H, d, J = 7.54 Hz) and δ 6.91 (1H, d, J = 7.54 Hz) indicated ortho-coupled aromatic H-atoms assignable to H-6′ and H-5′ in the B ring of flavonoid, respectively. In addition, doublet resonances at δ 6.41 (1H, s) and δ 6.21 (1H, s) for the H-8 and H-6 indicating the presence of the meta coupled connection. There was also a methoxy signal (δH 3.95, 3H, s) that is indicative of 3′-OCH_3_. Although an anomeric proton (δ 5.43, 1H, d) and some of the glycosidic protons overlapped (δ 3–4 ppm) in the ^1^H-NMR spectrum, in studying the ^13^C-NMR, the presence of five signals at δ 60–75 and a resonance at δ 102.1 (anomeric carbon of glucose), confirms the presence of glucose in the composition. The presence of a signal at δ 55.3 ppm in the ^13^C-NMR spectrum indicating a methoxy group which is confirmed by the presence of a singlet resonance at δ 3.95 (3H) in the ^1^H-NMR spectrum. In ^13^C -NMR, signals at δ 178.80, 164.60, 163.50, 160.00, 158.80, 130.90, 103.90, 98.40 and 93.30 corresponded to C-4, C-7, C-5, C-9, C-2, C-3, C-10, C-6 and C-8, respectively. Furthermore, signals at δ 151.90, 146.80, 122.30, 121.10, 114.60 and 112.9 corresponded to C-3′, C-4′, C-6′, C-1′, C-5′ and C-2′, respectively. Thus, compound 7 was identified unequivocally as isorhamnetin-3-*O*-beta-d-glucoside. Spectroscopic data were in good agreement with the literature data of the respective compound [37]. While isorhamnetin-3-*O*-beta-d-glucoside was previously reported from *A. halodendron*, [38] this is the first report for reporting this compound from *A. splendens*. Compound 8 was also isolated as a light yellowish powder from a 60% fraction as well. FABMS analyses (negative and positive ion modes) of compound 8 revealed the *pseudo*-molecular ions at m/z 623 [M−H]^−^ and 647 [M+Na]^+^, which was in agreement with the molecular formula C_28_H_31_O_16_ and C_28_H_32_O_16_Na, respectively. The ^1^H-NMR and ^13^C-NMR spectra of compound 8 were similar to those of compound 7, with the exception that one more rhamnose moiety has been regarded to this compound with four protons signals (δ 3.2–3.6), doublet resonance at δ 1.11 (3H, d, J = 6.04 Hz) and singlet resonance at δ 4.54 (1H, s), which is attributed to anomeric proton of H-1‴ of rhamnose moiety. Furthermore, the ^1^H-NMR spectra of compound 8 verified the existence of one isorhamnetin nucleus. Our claim has been supported by methoxy group shift (δ 3.95, 3H, s) in ^1^H-NMR as well as methoxy carbon signal (δ 55.30) at ^13^C-NMR; In ^1^H -NMR two aromatic spin systems, 7.95 (1H, d, J = 1.70 Hz), 7.64 (1H, dd, J = 8.40 and 1.70 Hz) and δ 6.95 (1H, d, J = 8.40 Hz) corresponded to H-2′, H-6′ and H-5′, respectively. Remaining peaks have been considered for 6.41 (1H, s) H-8 and 6.22 (1H, s) H-6.

The 1H-NMR spectrum of compound 3 showed the presence of one glucosyl moieties with characteristic doublet resonance at δ 5.24 (1H, d, J = 7.18 Hz) for the anomeric proton of β-glucose. In addition, the ^1^H-NMR spectra of compound 1 confirmed the presence of overlapping signals for four protons (δ 3.2–3.6 ppm), an anomeric proton (δ 4.54, 1H, s) and δ 1.11 (3H, d, 6.04) (methyl group of rhamnose), corresponding to a rhamnose moiety in the composition. In studying the ^13^C-NMR spectrum, the presence of five signals at δ 60–75 and a resonance at δ 102.7 (anomeric carbon of glucose), confirms the presence of glucose in the composition as well as the presence of four signals at δ 60–75, presence of a signal at δ 101.2 (anomeric carbon of rhamnose) and δ 16.5 (methyl group of rhamnose), was confirmed the presence of rhamnose in the composition. Furthermore, signals at δ 177.50, 164.60, 162.10, 157.30, 156.80, 134.30, 104.80, 98.10 and 93.50 corresponded to C-4, C-7, C-5, C-9, C-2, C-3, C-10, C-6 and C-8, respectively; as well as, signals at δ 149.80, 146.80, 122.70, 121.30, 115.30 and 113.40 corresponded to C-3′, C-4′, C-6′, C-1′, C-2′ and C-5′, respectively. On the basis of these data, the structure of 8 was elucidated as narcissin. Finding a literature review of previous works was compatible with our results [39]. While isorhamnetin-3-O-beta-D-glucoside was previously reported from *A. sublessingiana*, *A. absinthium*, *A. vulgaris* [40] and *A. incana*, [41] to the best of our knowledge, the respective compound was detected in this specie for the first time.

## Conclusion

In the current study, we reported the anti-proliferative and anti-microbial activities as well as chemical compositions of 60% and 40% SPE fractions which indicated maximum cytotoxic (against A549 and HT-29 cancerous cells) and inhibition zones against Gram-positive strains, respectively for the first time. Further analysis of both potent fractions afforded 5 flavonoids: narcisin (1), quercetin (2), luteolin (3), kaempferol (4) and genkwanin (5) from 60% SPE fraction as well as three known flavonoid glycosides, astragalin (6), isorhamnetin-3-O-beta-D-glucoside (7) as well as narcisin (8) from 40% SPE fraction for the first time from the *A. splendens*.

## Data Availability

The authors confirm that the data supporting the findings of this study are available within the article.
